# Speaking fluency and critical thinking among Egyptian EFL business students: a mixed-methods study

**DOI:** 10.3389/fpsyg.2026.1816706

**Published:** 2026-07-17

**Authors:** Israa Ismael, Xin Luo, Sen Li, Nasir Ali

**Affiliations:** 1 Department of Curriculum and Instructions, Faculty of Education, Shaanxi Normal University, Xi'an, China; 2 Department of Early Childhood, Faculty of Education, Shaanxi Normal University, Xi'an, China

**Keywords:** business students, critical thinking, Egypt, English as a Foreign Language, higher education, speaking fluency

## Abstract

The integration of speaking fluency (SF) and critical thinking (CT) is essential for effective academic and professional communication; however, these skills are often examined separately in English as a Foreign Language (EFL) research. Limited evidence exists regarding their interaction within Egyptian higher education, particularly in non-language faculties. This mixed-methods study investigated the relationship between SF and CT among 100 second-year Egyptian EFL business students at an intermediate (B1) level. Students completed a six-task performance-based test targeting multiple fluency skills (oral fluency, discourse management, suprasegmental features, interactive fluency, and formulaic expressions) and critical thinking skills (idea clarification, evidence provision, perspective integration, solution justification, and reflective reasoning). Students' performances were evaluated using analytic rubrics with strong internal consistency (SF α = 0.87; CT α = 0.90) and high inter-rater reliability (κ = 0.87). Descriptive statistics, Pearson correlation, and linear regression analyses were conducted. Results indicated moderate performance in both SF (*M* = 3.2, SD = 0.8) and CT (*M* = 3.0, SD = 0.9). A significant moderate positive correlation was found between SF and CT, *r*_(98)_ = 0.46, *p* < 0.001. Regression analysis showed that SF significantly predicted CT performance, explaining 21% of variance (*R*^2^ = 0.21). Qualitative findings from focus groups revealed speaking anxiety, overreliance on memorization, limited evaluative reasoning, and insufficient exposure to authentic communicative tasks. The findings suggest that speaking fluency supports the expression of critical thinking, although it does not ensure higher-order reasoning. The study highlights the need for integrated EFL instruction that connects oral communication practice with structured reasoning tasks to better develop both competencies.

## 1 Introduction

In both academic and professional contexts, the integration of speaking fluency (SF) and critical thinking (CT) is essential, as each skill reinforces and supports the other. Fluency enables coherent and effective oral communication, while critical thinking supports reasoning, argumentation, and problem-solving (Thuong, 2024). For example, an undergraduate student defending a project must present ideas fluently while justifying methodological choices, using evidence, and responding logically to questions (Irianti et al., 2024). This interdependence has also been highlighted in international research, where studies show that learners with stronger critical thinking skills tend to produce more coherent and structured spoken output in EFL and CLIL contexts (Bellaera et al., 2021). Similarly, recent work suggests that speaking performance in academic settings is shaped not only by linguistic accuracy but also by the ability to organize and defend ideas under communicative pressure (Salido et al., 2025).

In Egyptian higher education, however, assessment practices have traditionally emphasized written examinations, with comparatively limited focus on SF and CT performance (Abdul et al., 2024; Fisher, 2011; Kuchkorova and Isakova, 2025). This exam-oriented culture discourages the development of communicative competence, as students view English primarily as an academic subject rather than a practical communication tool (Mohanna, 2024). These patterns are consistent with broader findings in EFL contexts, where instructional systems that prioritize rote learning over communicative engagement tend to limit opportunities for developing higher-order thinking and spoken interaction skills (Hsu et al., 2022).

Additionally, a growing body of research has explored SF and CT through instructional interventions, yet these constructs are often examined separately. Despite increasing international attention to integrated skill development, empirical evidence documenting baseline performance across multidimensional fluency and reasoning skills within the same cohort remains limited, particularly in non-language faculties and business education contexts. Understanding how linguistic performance relates to cognitive reasoning in authentic academic speaking tasks is therefore necessary for advancing EFL assessment research (OECD, 2022).

This study presents empirical performance data on SF and CT among second-year EFL business students at the School of Business, Heliopolis University for Sustainable Development (HUSD), Egypt. Students' performances were assessed using a performance-based test designed to capture multiple dimensions of SF, including speech continuity, interactional competence, suprasegmental features, and formulaic expression, as well as CT, including clarification of ideas, evidence provision, perspective integration, problem-solving, and reflection on reasoning processes. By making these data publicly available, the dataset provides evidence for descriptive performance patterns, construct validation, and potential relationships between linguistic and cognitive measures in EFL higher education contexts.

## 2 Literature review

### 2.1 Speaking fluency in EFL contexts

SF involves more than speaking quickly; it refers to the ability to express ideas coherently, accurately, and meaningfully under real-time communicative pressure. Early definitions emphasized fluency as the continuity of speech and the ability to maintain meaning under communicative pressure (Fillmore, 1979), while later work highlighted the cognitive demands involved in converting thought into language efficiently (Lennon, 2000). Brumfit (1984) further broadened this view by linking fluency not only to speed or accuracy, but also to coherence, contextual appropriateness, and effective language use in spontaneous interaction.

Building on these theoretical foundations, recent research has operationalized speaking fluency into multiple measurable dimensions, including speech rate, pause frequency, smoothness, repair strategies, rhythm, and interactional competence (Cendra and Sulindra, 2022; Wang et al., 2024). These dimensions reflect the fact that fluency is not a single ability but a multidimensional construct involving both linguistic automaticity and interactional responsiveness.

Empirical studies in EFL contexts suggest that learners often show uneven development across these dimensions. For example, students may produce grammatically accurate speech but struggle with hesitation, breakdowns in interaction, or excessive pausing during spontaneous communication. Conversely, some learners may speak more quickly but with reduced clarity or coherence (Alhamad and Latif, 2023; Dogolsara et al., 2022). This indicates that fluency development is not solely dependent on linguistic knowledge, but also on the ability to retrieve and organize language in real time.

In the Egyptian higher education context, these challenges are often more pronounced due to limited exposure to interactive speaking practice and a strong focus on accuracy-based instruction. As a result, students may rely on memorized expressions or structured responses rather than spontaneous language production. This creates a gap between “knowing language rules” and “using language in real communication” (Falk et al., 2025).

Despite the growing body of research on speaking fluency, most studies have focused on general proficiency or isolated classroom interventions, with relatively limited attention given to how multiple fluency dimensions interact within specific academic cohorts, particularly in business-oriented EFL programs. This highlights the need for more context-sensitive research that captures fluency as a multidimensional performance rather than a single global score.

### 2.2 Critical thinking in EFL contexts

CT is widely recognized as essential in higher education for enabling students to analyze information, evaluate evidence, synthesize ideas, and draw reasoned conclusions (Dewey, 1910; Paul, 1988; Halpern, 2014; Ennis, 2015). It requires learners to move beyond surface comprehension toward deeper engagement, questioning assumptions and justifying viewpoints with evidence (Dwyer, 2017; Ennis, 2011). In academic settings, these abilities support interpretation, application of knowledge, and effective communication (Yaiche, 2021).

Within language education, research increasingly suggests that critical thinking is closely linked to communicative performance, particularly in speaking tasks. Learners who demonstrate stronger CT skills tend to produce more coherent arguments, better organized ideas, and more responsive interactional behavior during oral communication (Bellaera et al., 2021; Salido et al., 2025). This suggests that speaking performance is not purely linguistic, but also depends on the ability to structure reasoning and maintain conceptual clarity during real-time production. Similarly, Irianti et al. (2024) found that learners with higher CT engagement are more likely to participate actively in discussions, evaluate peer contributions, and make informed communicative decisions.

However, the development of CT in EFL contexts remains uneven and often constrained by both cognitive and instructional factors. One key challenge is the overwhelming exposure to digital information, which may encourage surface-level processing rather than deeper evaluation and reflection (D'Northwood and Rattray, 2024). In addition, linguistic limitations such as restricted vocabulary and limited syntactic control may prevent learners from fully expressing complex reasoning, even when such reasoning exists at the conceptual level.

From an instructional perspective, CT is often implicitly included in curricula but not explicitly taught as a structured skill. Hsu et al. (2022) note that many teachers attempt to promote critical thinking without formal training in CT pedagogy, which results in classroom practices that are not systematically aligned with CT development. In many cases, teacher talk dominates classroom interaction, limiting opportunities for students to engage in extended reasoning, questioning, or argumentation.

Moreover, students' conceptual understanding of CT may itself be limited. Previous research indicates that some learners interpret critical thinking narrowly as simply “disagreeing with others,” which may discourage them from engaging in constructive evaluation or discussion (Yuan et al., 2022). This misconception can reduce the quality of classroom interaction and limit the development of deeper analytical skills.

In EFL contexts, particularly in settings where English is learned as a foreign language, CT development is further constrained by the lack of structured opportunities for analytical discourse. Many programs prioritize content coverage and language accuracy over reasoning, evaluation, and argumentation (Awaad et al., 2025; Wahab and Terasne, 2020). As a result, learners may develop declarative knowledge without sufficient practice in applying reasoning skills in spoken academic communication.

Taken together, existing literature suggests that critical thinking is essential for academic communication, but its development in EFL contexts is influenced by linguistic proficiency, instructional practices, and learners' prior educational experiences. Despite this recognition, there is still limited empirical research that examines how critical thinking is actually demonstrated during authentic spoken performance tasks, particularly when integrated with speaking fluency assessment in the same cohort.

### 2.3 The Egyptian higher education context

In the Egyptian higher education context, English functions as the primary foreign language across multiple academic and professional domains, including business, science, media, tourism, and technology. Despite its importance, EFL learners continue to face persistent linguistic, pedagogical, psychological, and institutional constraints that collectively affect their ability to communicate effectively and to engage in higher-order thinking during English use (Alhamad and Latif, 2023).

From a linguistic perspective, one of the central challenges arises from structural differences between Arabic and English. As Arabic belongs to the Semitic language family and English to the Germanic family, the two systems differ significantly in grammar, syntax, and phonology. These differences often lead to first-language interference, which affects sentence construction and spoken accuracy (Latif, 2024). Vocabulary limitations further restrict students' communicative ability, as many learners rely heavily on memorized lexical chunks rather than flexible language use. Le et al. (2024) report that students frequently remain passive during lectures because they lack sufficient lexical resources to express their ideas. Similarly, Wang et al. (2024) found that reliance on rote memorization of fixed expressions often results in hesitations, pauses, and fragmented speech during real-time communication.

Pronunciation also presents a persistent barrier. Several English phonemes, such as /p/, /v/, and /η/, do not exist in Arabic, leading to substitution patterns that reduce intelligibility (Syed and Abdelrady, 2022). For instance, learners may replace /p/ with /b/ or /θ/ with /s/, which can affect clarity in extended speech (Abdelreheem, 2023). Alongside phonological challenges, grammatical awareness tends to be treated as a written rather than spoken concern. Cabezas and Beltran (2021) note that learners often prioritize grammatical accuracy in writing tasks, which leads to hesitation and self-correction in speaking as they attempt to apply explicit rules during spontaneous communication.

Psychological factors further intensify these linguistic difficulties. Many learners experience speaking anxiety, low confidence, and reluctance to participate in oral interaction due to fear of making mistakes or being negatively evaluated (Alhamad and Latif, 2023). Sahmal et al. (2024) similarly found that students often avoid speaking in class because they fear embarrassment or criticism from peers and instructors. In some cases, perceived gaps in proficiency between students also contribute to anxiety and reduced willingness to participate (Al Zahrani and Elyas, 2017). These affective barriers limit opportunities for practice, which in turn slows the development of both fluency and communicative confidence.

Pedagogical and institutional conditions also play a significant role. Large class sizes and teacher-centered instructional approaches dominate many Egyptian university classrooms, limiting opportunities for meaningful student interaction (Mekheimer and Fageeh, 2024). In such environments, teachers often control discourse, leaving limited space for students to engage in extended speaking practice or exploratory discussion. In addition, assessment practices remain largely summative and recall-oriented rather than performance-based or inquiry-driven (Khodary, 2020). As a result, students tend to focus on producing correct answers rather than developing reasoning, argumentation, or communicative flexibility.

Furthermore, curricular and material constraints reinforce these patterns. Many textbooks used in Egyptian universities are not fully aligned with learners' linguistic level or communicative needs, as they are often designed for international contexts (Attia and Algazo, 2025). Limited institutional resources and budget constraints also reduce opportunities for professional development and interactive teaching innovation (Kaoud et al., 2021). In some cases, this leads to a reliance on static teaching materials rather than communicative or task-based learning approaches. Additionally, the post-COVID shift toward online assessment has raised concerns about academic integrity and consistency in evaluation practices due to limited monitoring systems and uneven digital literacy.

Taken together, these linguistic, psychological, pedagogical, and institutional factors suggest that English learning in the Egyptian context is often characterized by limited opportunities for spontaneous interaction and analytical language use. While these challenges have been widely discussed in previous literature, there remains a lack of empirical evidence examining how they manifest simultaneously in students' speaking fluency and critical thinking performance within the same communicative tasks.

More specifically, existing studies tend to examine either speaking fluency or critical thinking separately, or rely on controlled tasks that do not fully capture authentic classroom performance. As a result, the interaction between linguistic fluency and cognitive reasoning processes in real EFL academic speaking contexts remains underexplored (Ban et al., 2023; Dicataldo and Dipace, 2025; Salido et al., 2025). In particular, there is limited multidimensional evidence addressing how learners perform across integrated dimensions such as speech continuity, interactional competence, and suprasegmental features on the one hand, and idea clarification, evidence use, perspective integration, and reflective reasoning on the other.

Therefore, the present study responds to this gap by examining second-year EFL business students at Heliopolis University for Sustainable Development (HUSD), Egypt, using an explanatory sequential mixed-methods design. The study first investigates performance patterns in speaking fluency and critical thinking through a structured performance-based assessment. Subsequently, qualitative focus group discussions were conducted to explore underlying challenges influencing students' performance and learning experiences. Accordingly, the study was guided by the following research questions:

**RQ1**. How do second-year EFL business students perform across different dimensions of speaking fluency?
**RQ2**. How do second-year EFL business students perform across different dimensions of critical thinking?
**RQ3**. What is the relationship between speaking fluency and critical thinking performance among Egyptian EFL business students?
**RQ4**. What linguistic, cognitive, and pedagogical factors influence second-year EFL business students' speaking fluency and critical thinking performance as revealed through qualitative focus group analysis?

## 3 Methods

### 3.1 Study design

The study employed an explanatory sequential mixed-methods design (Creswell and Plano Clark, 2018; Tashakkori and Teddlie, 2010). This design was selected because quantitative findings could identify performance patterns and relationships between speaking fluency and critical thinking, whereas qualitative data were required to explain the underlying linguistic, cognitive, and instructional factors influencing students' performance. In the first phase, quantitative data were collected through a performance-based assessment designed to evaluate multiple dimensions of SF and CT. In the second phase, semi-structured focus group discussions were conducted with a subset of participants to explore students' perceptions, challenges, and learning experiences associated with the assessed skills. The integration of quantitative and qualitative findings enabled methodological triangulation and provided a more comprehensive understanding of students' communicative and reasoning abilities than either approach alone.

The performance-based assessment was grounded in established theoretical frameworks of speaking fluency and critical thinking. The critical-thinking component was informed by Facione's (1990) Delphi Report and Ennis's (2011), 2015) conceptualization of critical thinking, particularly the skills of evaluating information, using evidence, considering alternative perspectives, drawing reasoned conclusions, and reflecting on thinking processes. The speaking-fluency component was informed by the Common European Framework of Reference for Languages (CEFR) descriptors for spoken production and interaction, as well as research emphasizing speech continuity, discourse management, interactional competence, repair strategies, and suprasegmental features. The assessment was therefore designed to elicit authentic spoken performances requiring students to demonstrate both linguistic fluency and higher-order reasoning in realistic communicative situations.

### 3.2 Participants

One hundred second-year intermediate-level (B1) EFL students from the School of Business at Heliopolis University for Sustainable Development (HUSD), Egypt, participated in the performance-based assessment. The sample represented the entire cohort of second-year business students enrolled during the data-collection period, thereby minimizing sampling bias and ensuring representation of the target population within the institutional context. Students participated in seven face-to-face assessment sessions, each comprising approximately 13–15 participants and lasting approximately 2.5 h. Prior to participation, students received information regarding the study objectives and procedures and provided informed consent.

In addition, 20 students participated in follow-up focus group discussions. Unlike purely convenience-based selection, a purposeful maximum variation sampling strategy was applied based on quantitative performance results from the SF and CT assessments. Specifically, after scoring and ranking participants, students were categorized into three performance groups (high, moderate, and low achievers) according to their composite SF and CT scores. From these groups, 20 participants were selected to ensure representation across performance levels. This approach was adopted to ensure that qualitative data captured diverse learner experiences and provided explanatory insights into the full range of performance observed in the quantitative phase. Participants were divided into three focus groups consisting of 6–7 students each, and each session lasted approximately 60 minutes.

### 3.3 Instruments

#### 3.3.1 Performance-based assessment

A researcher-developed performance-based assessment consisting of six authentic speaking tasks was designed to evaluate both SF and CT (Appendix A). The tasks were developed to simulate realistic communicative situations that required participants to express ideas, justify opinions, evaluate information, and solve problems while maintaining fluent oral communication.

All participants completed the same assessment under standardized conditions. Students were allowed brief preparation time before each task and could make notes for planning purposes; however, they were encouraged to speak spontaneously rather than read prepared responses. All performances were audio-recorded for subsequent rating and analysis.

The six tasks were administered as follows:

- **Hypothetical reasoning (“What if?”):** required students to respond to personal hypothetical scenarios (e.g., living without internet for 1 month or becoming invisible for a day). Students were given approximately 1 min for preparation and up to 2 mins for their response. This task primarily assessed speech rate, pause management, idea clarification, and solution justification.
- **Trending news discussion:** required students to select a current news item from online, print, or social media sources, identify its main idea, summarize it, and provide a personal opinion. Students received approximately 2 mins of preparation time and up to 3 mins for discussion. This task assessed discourse management, perspective integration, and evidence-based reasoning.
- **A debate:** required students to select one controversial statement, indicate agreement or disagreement, and defend their position. Participants were given approximately 2 mins for preparation and up to 3 mins for their response. The task assessed argument construction, response to counterarguments, speech continuity, and formulaic language use.
- **Real-life problem-solving:** presented practical scenarios requiring students to propose and justify solutions. Students received approximately 2 mins of preparation and up to 3 mins to explain their responses. This task evaluated problem-solving, justification of decisions, logical sequencing, and maintenance of fluent speech.
- **Video response:** required students to watch a short authentic video (“Teens React to Giving Up Social Media for a Week”), which was played once for all participants. Students were allowed to take notes while viewing the video and were subsequently asked a series of open-ended questions assessing interpretation, evidence use, reflection, and evaluation. Participants were given approximately 1 min to organize their notes before responding. This task primarily assessed analytical thinking, evidence-based reasoning, and suprasegmental features such as rhythm, stress, and intonation.
- **Invent an application:** required students to design and describe a mobile application, explain the problem it addressed, and justify its potential value. Students were given approximately 3 mins for planning and up to 3 mins for presentation. This task assessed idea development, decision-making, reasoning, coherent organization, and natural spoken delivery.

Additionally, Table 1 summarizes the performance-based assessment tasks and the speaking fluency and critical thinking skills targeted by each task.

**Table 1:** Performance-based assessment tasks and targeted speaking fluency and critical thinking skills.

Task	Description	Speaking fluency skills	Critical thinking skills
Hypothetical reasoning (“What If?”)	Respond to personal hypothetical scenarios	Speech rate, pause management	Idea clarification, solution proposal, and justification
Trending news discussion	Summarize and discuss a current news item	Discourse management, coherent sequencing	Perspective integration, evidence-based reasoning
Debatable statement defense	Defend a position on a controversial issue	Speech continuity, formulaic expressions, and response to counterarguments	Argument evaluation, justification of position
Real-life problem solving	Generate solutions to practical scenarios	Steady speech flow, logical progression of ideas	Multi-perspective problem solving, justification
Video response	Watch a video and answer analytical questions	Rhythm, stress, intonation, and clarity	Analysis, evidence use, and reflection
Invent an application	Design and explain a mobile application	Coherent organization, natural delivery, and formulaic language	Idea generation, decision-making, and reasoning

#### 3.3.2 Analytic rubrics and scoring procedures

Students' performances were evaluated using separate analytic rubrics for SF and CT (Appendices B, C). The rubrics were developed based on established theoretical frameworks and existing assessment literature and subsequently refined through expert review. The critical-thinking rubric was adapted from the work of Facione (1990) and (Ennis 2011, 2015) and assessed six broad dimensions: evaluating information, using evidence, organizing ideas logically, asking purposeful questions, responding to counterarguments, and drawing reasoned conclusions. These dimensions were operationalized through ten observable performance indicators.

The speaking-fluency rubric was informed by CEFR descriptors for spoken interaction and production and assessed four broad dimensions: speech flow, pause management, repair fluency, and intonation for smoothness. These dimensions were operationalized through eight observable performance indicators. All rubric criteria were scored using a four-point scale ranging from 1 (Ineffective) to 4 (Outstanding). Individual criterion scores were averaged to generate overall SF and CT scores for each participant. The complete rubric descriptors are provided in Appendices B, C to facilitate transparency and replication.

Content validity was established through expert review by five specialists in Applied Linguistics who evaluated the alignment between tasks, constructs, and rubric descriptors. Internal consistency was high for both instruments (SF α = 0.87; CT α = 0.90). To minimize construct contamination, speaking fluency and critical thinking were operationalized as separate constructs and evaluated using independent analytic rubrics. For each participant, raters completed a speaking-fluency rubric and a critical-thinking rubric separately. Speaking-fluency ratings focused exclusively on observable features of oral delivery, including speech flow, pause management, repair strategies, and intonation. In contrast, critical-thinking ratings focused on the quality of reasoning, including relevance of information, use of evidence, organization of ideas, consideration of alternative perspectives, and justification of conclusions. Grammatical accuracy, pronunciation, and lexical sophistication were not included in the critical-thinking rubric, while the strength of arguments and quality of reasoning were not included in the speaking-fluency rubric. This scoring procedure was adopted to reduce construct overlap and ensure that linguistic performance and reasoning performance were evaluated as distinct dimensions.

##### 3.3.2.1 Rater training and reliability

Two PhD-qualified lecturers from the university's Applied Linguistics Department, each with more than 10 years of experience in language assessment and EFL instruction, independently evaluated all recorded performances. Prior to scoring, both raters reviewed the assessment tasks, scoring rubrics, and performance descriptors to establish a shared understanding of the evaluation criteria and to ensure clear differentiation between linguistic performance indicators and critical-thinking indicators. Speaking fluency and critical thinking were scored separately to minimize construct overlap. Fluency ratings focused exclusively on features such as speech continuity, pause management, repair strategies, and intonation, whereas critical-thinking ratings focused on evidence use, reasoning quality, argument development, perspective integration, and justification. Inter-rater reliability analysis demonstrated strong agreement between raters (Cohen's κ = 0.87).

#### 3.3.3 Focus group discussions

Following completion of the quantitative phase, semi-structured focus group discussions were conducted to explore factors influencing students' speaking fluency and critical-thinking performance. The interview guide included questions addressing speaking anxiety, preparation strategies, confidence in expressing opinions, use of evidence and reasoning, classroom participation experiences, and perceived challenges associated with English communication.

All answers were audio-recorded with participants' consent and transcribed using intelligent verbatim transcription. The qualitative phase was intended to provide explanatory insight into patterns identified during quantitative analysis and to strengthen interpretation of the overall findings through methodological triangulation.

### 3.4 Data analysis

Students' performances were independently scored by two raters from the university's Languages Department. Inter-rater reliability demonstrated strong agreement (Cohen's κ = 0.87), and internal consistency was high for both rubrics (SF α = 0.87; CT α = 0.90).

Quantitative data were analyzed using SPSS (Version 25). Descriptive statistics (means, standard deviations, minimum, and maximum scores) were calculated for all SF and CT dimensions. Prior to inferential analyses, assumptions for Pearson correlation and linear regression were examined. Normality was assessed through visual inspection of histograms and Q–Q plots, as well as skewness and kurtosis values, which were within acceptable ranges (±1.5). Linearity was examined using scatterplots, indicating an approximately linear relationship between SF and CT. Homoscedasticity was evaluated through standardized residual plots, which showed no systematic pattern. Influential cases were assessed using Cook's Distance values, all of which were below 1. Standardized residuals indicated no extreme outliers (all within ±3.0).

Pearson correlation analysis was conducted to examine the relationship between speaking fluency and critical thinking. A simple linear regression analysis was then performed to determine the extent to which SF predicted CT performance. Regression diagnostics confirmed that the assumptions of linearity, normality of residuals, homoscedasticity, and independence of errors were adequately met.

Qualitative data were analyzed using a hybrid inductive–deductive thematic analysis approach. The deductive component was guided by the study's conceptual framework of speaking fluency and critical thinking, while inductive coding allowed for the emergence of new patterns from the data. Transcripts were read repeatedly to ensure familiarization, followed by open coding that generated 53 initial codes. These codes were then organized into 11 categories and further refined into 4 overarching themes through iterative comparison.

To enhance trustworthiness, a second coder independently reviewed approximately 40% of the transcripts. Discrepancies were discussed until consensus was reached. In addition, peer debriefing with two Applied Linguistics specialists was conducted to validate theme structure and interpretation. Final themes were agreed upon collaboratively. Quotations were selected to reflect recurring patterns across participants rather than isolated responses.

## 4 Results

### 4.1 Quantitative results

Students' performances were evaluated using analytic rubrics on a five-point scale (1 = Poor, 5 = Excellent). The performance of second-year EFL students (*N* = 100) on speaking fluency (SF) and critical thinking (CT) tasks revealed moderate performance across both linguistic and cognitive domains. Students' SF was strongest in Automaticity/Formulaic Expressions (*M* = 3.8, SD = 0.7), followed by Discourse Management (*M* = 3.5, SD = 0.8) and Oral Fluency (*M* = 3.2, SD = 0.9), whereas Suprasegmental Features (*M* = 2.9, SD = 1.0) and Interactive Fluency (*M* = 2.8, SD = 1.1) were the weakest, reflecting challenges in spontaneous interaction and turn-taking (Table 2).

**Table 2:** Speaking fluency dimensions.

Dimension	Mean	SD	Min	Max
Oral fluency	3.2	0.9	1	5
Discourse management	3.5	0.8	2	5
Suprasegmental features	2.9	1.0	1	5
Interactive fluency	2.8	1.1	1	5
Formulaic expressions	3.8	0.7	2	5

For CT dimensions, students performed relatively better in Providing Evidence (*M* = 3.3, SD = 0.8) and Clarifying Information and Ideas (*M* = 3.1, SD = 0.9). Lower scores were recorded for Integrating Perspectives (*M* = 2.7, SD = 1.0) and Reflecting on Thinking Processes (*M* = 2.8, SD = 1.0), suggesting weaknesses in evaluative reasoning and metacognitive awareness. Proposing and Justifying Solutions showed moderate performance (*M* = 3.0, SD = 0.9; Table 3).

**Table 3:** Critical thinking dimensions.

Dimension	Mean	SD	Min	Max
Clarify information and ideas	3.1	0.9	1	5
Provide evidence	3.3	0.8	2	5
Integrate different perspectives	2.7	1.0	1	5
Propose and justify solutions	3.0	0.9	1	5
Reflect on thought process	2.8	1.0	1	5

Preliminary assumption testing supported the use of Pearson correlation and linear regression analyses. Visual inspection of histograms and Q–Q plots indicated that SF and CT scores approximated normal distributions. Skewness and kurtosis values were within acceptable limits. Scatterplot analysis demonstrated a linear relationship between the two variables, while examination of residual plots indicated homoscedasticity. No influential cases were detected, as Cook's Distance values remained below 1.0, and standardized residuals fell within acceptable limits (±3.0). Therefore, Pearson correlation and simple linear regression were considered appropriate for examining the relationship between SF and CT performance. Overall, the mean SF score (*M* = 3.20, SD = 0.80) slightly exceeded the mean CT score (*M* = 3.00, SD = 0.90).

#### 4.1.1 Relationship between SF and CT

Pearson correlation analysis revealed a statistically significant moderate positive relationship between speaking fluency and critical thinking performance, *r*_(98)_ = 0.46, *p* < 0.001, indicating that students who demonstrated higher levels of speaking fluency tended to exhibit stronger critical-thinking performance. A simple linear regression analysis was conducted to examine whether speaking fluency significantly predicted critical-thinking performance. The regression model was statistically significant, *F*_(1, 98)_ = 26.18, *p* < 0.001, explaining approximately 21% of the variance in CT scores (*R*^2^ = 0.21, Adjusted *R*^2^ = 0.20).

Speaking fluency emerged as a significant predictor of critical thinking, *B* = 0.52, SE = 0.10, β = 0.46, *t* = 5.12, and *p* < 0.001, indicating that a one-unit increase in speaking fluency was associated with an estimated 0.52-point increase in critical-thinking performance. The 95% confidence interval for the unstandardized coefficient ranged from 0.32 to 0.72, suggesting a stable positive effect. Inspection of regression diagnostics indicated that the assumptions of linearity, homoscedasticity, normality of residuals, and independence of errors were adequately met. The Durbin–Watson statistic was 1.94, indicating no evidence of autocorrelation among residuals. Detailed regression coefficients, standard errors, confidence intervals, and model statistics are presented in Table 4.

**Table 4:** Simple linear regression predicting critical thinking from speaking fluency.

Predictor	*B*	SE	β	*t*	*p*	95% CI
Constant	1.34	0.29	–	4.62	<0.001	[0.76, 1.92]
Speaking Fluency	0.52	0.10	0.46	5.12	<0.001	[0.32, 0.72]

Model statistics: R^2^ = 0.21, Adjusted R^2^ = 0.20, F_(1, 98)_ = 26.18, p <0.001, Durbin–Watson = 1.94.

As shown in Table 4, speaking fluency was a statistically significant positive predictor of critical-thinking performance, accounting for approximately one-fifth of the variance in students' CT scores.

### 4.2 Qualitative results

Thematic analysis revealed four major themes: (1) linguistic barriers to speaking fluency, (2) speaking anxiety and self-confidence, (3) limitations in critical-thinking performance, and (4) instructional and contextual influences on learning. Together, these themes provided explanatory insights into the quantitative findings.

#### 4.2.1 Theme 1: linguistic barriers to speaking fluency

The most frequently reported challenge involved difficulties maintaining smooth and spontaneous speech. Students described frequent hesitation, excessive pausing, lexical retrieval problems, and reliance on filler expressions. Similar patterns were observed during the performance-based assessment, where participants often interrupted their speech while searching for vocabulary or reformulating ideas.

One student stated:

“*I know what I want to say, but sometimes I cannot find the English words quickly, so I stop and think before continuing*.”

Another participant explained:

“*When I forget a word, I switch to Arabic because I don't know another way to explain it*.”

These findings help explain the relatively low scores obtained in interactive fluency and suprasegmental features during the quantitative phase.

#### 4.2.2 Theme 2: speaking anxiety and self-confidence

A second recurring theme involved anxiety associated with oral communication. Participants frequently reported fear of making grammatical mistakes, being judged by peers, or receiving negative evaluation from instructors.

One student commented:

“*Sometimes I cannot breathe properly when speaking because I am afraid that my classmates will laugh if I make a mistake*.”

Another participant stated:

“*Even if I know the answer, I become nervous when everyone is listening to me*.”

Students indicated that anxiety often increased hesitation, reduced participation, and encouraged reliance on memorized responses rather than spontaneous communication.

#### 4.2.3 Theme 3: limitations in critical-thinking performance

Participants also described difficulties generating ideas, evaluating alternative perspectives, and justifying opinions. These findings were consistent with the lower quantitative scores observed in perspective integration and reflective reasoning.

For example, when discussing the application-design task, one participant stated:

“*Honestly, there was nothing in my mind, even in Arabic. I did not know what idea I could talk about*.”

Similarly, several students struggled to propose alternative titles during the video-response activity and frequently responded:

“*I don't have any title in my mind*.”

These responses suggested that some difficulties reflected challenges in idea generation and reasoning rather than language limitations alone.

#### 4.2.4 Theme 4: instructional and contextual influences

Participants frequently attributed their difficulties to previous learning experiences and assessment practices. Many reported that English courses primarily emphasized memorization, written examinations, and reproduction of information rather than discussion, argumentation, or problem-solving.

One student explained:

“*Most exams focus on memorizing information, so we study for the test rather than learning how to discuss ideas*.”

Another participant commented:

“*We rarely practice speaking in class, so when we are asked to explain our opinions, it feels difficult*.”

Students also reported limited exposure to authentic English input outside the classroom, which reduced opportunities to develop both fluency and critical engagement with ideas. Overall, the qualitative findings provided explanatory support for the quantitative results. Difficulties related to vocabulary retrieval, anxiety, limited spontaneous communication practice, and restricted opportunities for analytical discussion appeared to contribute to weaker performance in interactive fluency, suprasegmental features, perspective integration, and reflective reasoning. The qualitative data therefore helped explain not only what performance patterns emerged in the quantitative phase but also why those patterns may have occurred.

## 5 Discussion

The findings provide empirical insight into the development of SF and CT among Egyptian EFL university students. Quantitative results indicated moderate performance across both domains, with relatively stronger performance in formulaic expression and discourse management compared to interactive fluency and suprasegmental features. These patterns suggest that students may rely more on memorized or structured linguistic resources rather than spontaneous communicative production. This observation is consistent with prior research showing that EFL learners often prioritize lexical and grammatical accuracy over real-time interactional competence (Wang et al., 2024; Falk et al., 2025).

The qualitative findings further explain these performance patterns by revealing behavioral, cognitive, and affective barriers to oral communication. Students' frequently demonstrated hesitation, excessive pausing, code-switching to Arabic when lexical retrieval failed, and reliance on prepared notes or externally generated content. Such behaviors reflect limited automaticity in language processing and increased cognitive load during spontaneous speech production. According to fluency theory, speech production becomes more efficient when linguistic retrieval operates with reduced conscious monitoring, allowing ideas to be expressed smoothly under communicative pressure (Fillmore, 1979; Lennon, 2000; Brumfit, 1984).

CT performance showed comparable constraints. Although, students were able to provide basic clarification of ideas and evidence, higher-order reasoning skills such as perspective integration, justification of arguments, and reflective evaluation were less developed. This pattern aligns with previous research suggesting that EFL students often experience difficulty translating cognitive reasoning into coherent spoken output due to linguistic limitations and instructional practices emphasizing recall-based learning (Paul, 1988; Halpern, 2014; Ennis, 2015; Dwyer, 2017).

The moderate positive correlation between SF and CT supports theoretical assumptions that linguistic fluency and cognitive reasoning are interrelated but represent partially distinct dimensions of communicative competence. Fluency facilitates the expression of thought by reducing production constraints, while critical thinking provides structural organization and argumentative depth to spoken communication. However, the relationship is not absolute, indicating that improvement in linguistic proficiency does not automatically guarantee development of higher-order reasoning ability (Tuychiev, 2023). This finding supports cognitive-linguistic perspectives suggesting that communication competence emerges from the interaction between language processing efficiency and conceptual reasoning capacity.

Nevertheless, these findings should be interpreted with caution. Because the study employed a cross-sectional correlational design, no causal relationship can be inferred between speaking fluency and critical thinking. Although students with higher speaking-fluency scores tended to obtain higher critical-thinking scores, it cannot be concluded that improvements in speaking fluency lead to improvements in critical thinking, or vice versa. Alternative explanations are also possible. For example, students with higher overall English proficiency may have been better able to articulate their reasoning during oral tasks, resulting in higher performance on both constructs. Similarly, prior educational experiences, confidence in oral communication, motivation, and familiarity with performance-based assessment tasks may have influenced students' scores. Future longitudinal and experimental studies are needed to examine the direction and nature of the relationship between speaking fluency and critical thinking more closely.

From a pedagogical perspective, the findings highlight structural challenges in Egyptian EFL higher education. Limited instructional time allocated to English courses, large class sizes, and assessment systems emphasizing written examinations may restrict opportunities for authentic oral communication. In many cases, classroom interaction is teacher-centered, reducing student participation and discouraging exploratory reasoning. Previous research has similarly reported that recall-based evaluation systems may limit the development of analytical and reflective thinking skills (Khodary, 2020).

The study also highlights the potential value of integrating speaking practice with critical thinking tasks within business education contexts. English for Specific Purposes instruction can be strengthened by incorporating authentic professional communication scenarios such as negotiation, case analysis, and decision-making discussions (Rajprasit et al., 2022). Low-cost digital learning tools may further support interactive learning without requiring advanced technological infrastructure. Platforms such as TED-Ed and Voice of America Learning English can provide graded authentic input for listening comprehension, summarization, and discussion tasks. Collaborative brainstorming and argument visualization activities can be supported using open educational tools including Padlet, Edpuzzle, Mentimeter, and Jamboard (Dias-Oliveira et al., 2024; Meirbekov et al., 2022).

Rather than restricting the use of artificial intelligence tools, structured AI literacy activities may help students critically evaluate machine-generated content and justify revisions through oral explanation. Such approaches may promote both metacognitive awareness and communicative autonomy in EFL learning environments.

### 5.1 Limitations and directions for future research

Although separate analytic rubrics were used to distinguish speaking fluency from critical thinking, both constructs were assessed through the same oral tasks. Consequently, some degree of interaction between linguistic performance and reasoning performance cannot be completely ruled out. In addition, the study employed a cross-sectional correlational design; therefore, causal conclusions regarding the relationship between speaking fluency and critical thinking cannot be drawn. Other variables that were not directly measured, such as overall English proficiency, prior educational experiences, communication confidence, and familiarity with oral assessment tasks, may also have contributed to performance in both domains. Future studies may benefit from incorporating additional measures of critical thinking that are less dependent on oral language production and from using longitudinal or experimental designs to examine causal relationships.

### 5.2 Conclusion

The present study provides empirical evidence on the relationship between speaking fluency and critical thinking among Egyptian EFL business students. The results indicate moderate development in both domains and a significant positive association between speaking fluency and critical-thinking performance. However, this relationship should be interpreted as correlational rather than causal, and additional research is needed to clarify the direction and underlying mechanisms of the association. These findings emphasize the importance of designing EFL curricula that simultaneously support fluency development and critical thinking training within authentic academic and professional communication contexts.

## Data Availability

The original contributions presented in the study are included in the article/Supplementary material, further inquiries can be directed to the corresponding author.
