# Supplementary material for: Speaking fluency and critical thinking among Egyptian EFL business students: a mixed-methods study
Source: Front Psychol. 2026 Jul 17;17:1816706. doi: 10.3389/fpsyg.2026.1816706 (PMC13424477; doi:10.3389/fpsyg.2026.1816706)
Supplement: Supplementary file 1 [file Supplementary_file_1.pdf]

Supplementary Materials

Appendix A

A Performance-Based Assessment of Speaking Fluency and Critical Thinking for Egyptian Business EFL Undergraduates

Section A: Speaking Activation Activity

Title: *What If?*

Instructions: Choose one question and answer it within 2 minutes.

- What if you had to live without internet for a month? How would you cope?
- If you could change something about your past, what would you change?
- If you were invisible for one day, what would you do?
- Where would you live if you could live anywhere in the world and why?

Section B: Trends on Media

Instructions: Choose a piece of news (online, newspaper, or social media) and:

1. Identify the main idea.
2. Summarize it in your own words.
3. Give your personal point of view.

Section C: Opinion Task

Instructions: Choose one statement from the list below. State whether you agree or disagree and explain why (3 minutes).

Online learning is better than face-to-face.	Smartphones should be banned at schools.	People who live in rural areas are less educated.

Students in private schools are better than those in public ones.	Artificial intelligence will replace teachers in the future.	Technology in the classroom is more distracting than helpful.
Teenagers are irresponsible and always on their phones.	Uniforms should be banned.	Books should be replaced with tablets.
Schools should teach life skills, like coding, rather than traditional subjects.	Mixed-gender classes should be banned in schools.	Women should serve in the military for one year like men.
Learning a foreign language should be optional, not mandatory.	Exams should be banned.	Once physical punishment is banned, students no longer care about learning.

Section D: Real-Life Scenario Ideas

Instructions: Choose one scenario and explain how you would solve it.

- You are about to give an important presentation. The night before, your laptop crashes and you lose your slides and notes.
- Your friend repeatedly borrows your things without asking. What’d you do?
- You want to start a small business, but your family disagrees. How would you persuade them?
- You are leading a team project, but some members are not contributing. How would you address the issue?
- You missed an important flight due to traffic. How would you handle the situation?
- You need to prepare for multiple exams happening in the same week. How would you plan your study schedule effectively?

Section E: Listening Activity

Title: *Teens React to Giving Up Social Media for a Week*

Pre-Listening Questions:

- Look at the title and predict what the video will be about.
- What would you expect to happen if teenagers gave up social media for a week?

Video Link: https://www.youtube.com/watch?v=OsLgTbQmBXQ

Post-Listening Questions:

1. What were the most significant changes the teens reported during their week without social media?
2. What are some possible negative consequences of giving up social media that the teens didn’t mention?
3. Which examples or stories the teens shared seem most convincing to you? Why?
4. Would you dare to do the same experiment? What obstacles would you face?
5. Suggest a new title for this video.

Section F: Invent an Application

Instructions: Invent a mobile application and cover the following points:

1. What is the name of the app?
2. Design a logo (hand-drawn or digital).
3. What problem does it solve?
4. What will you do with the money you earn from it?

Appendix (B)

Critical Thinking Skills Scoring Rubric

Critical Thinking Skills	Outstanding (4)	Effective (3)	Adequate (2)	Ineffective (1)
**A. Evaluating Relevant Information from Multiple Sources**				
A1. Selecting relevant information	Most information is relevant; irrelevant details are rare and do not affect the response (e.g., 0–1 minor off-topic points).	Information is mostly relevant, some minor irrelevant details present (e.g., 2 points).	Some relevant information, irrelevant or redundant details noticeable (e.g., 3–4 points).	Most information is irrelevant, off-topic, or distracting (e.g., 5+ points).
A2. Distinguishing main ideas from supporting details	Clearly identifies main ideas and consistently separates them from details	Mostly identifies main ideas; distinction from details occasionally unclear	Main ideas partially identifiable; mixed with details	Main ideas not identifiable or completely mixed with details
**B. Using Evidence to Support Spoken Ideas**				
B1. Selecting appropriate evidence	Evidence relevant and strengthens claim (3+ examples/facts)	Mostly relevant; minor gaps (2 examples/facts)	Partially relevant or weak support (1 example/fact)	Evidence absent or unrelated
B2. Explaining evidence relevance	Clear and explicit explanation of evidence relevance, showing how it supports the claim	Partial or implicit explanation of evidence relevance	Minimal or vague explanation of evidence relevance	Absence of explanation of evidence relevance
**C. Organizing Ideas Logically**				

Critical Thinking Skills	Outstanding (4)	Effective (3)	Adequate (2)	Ineffective (1)
C1. Logical organization of ideas	Ideas consistently organized (introduction–development–conclusion) throughout response.	Organization mostly logical; minor sequencing issues	Organization uneven; ideas sometimes disconnected	No clear organizational structure
**D. Asking Relevant and Purposeful Questions**				
D1. Asking clarification and follow-up questions	Consistent asking of clarification and follow-up questions that explore causes, consequences, or alternative perspectives	Frequent asking of clarification and follow-up questions, with partial exploration of causes, consequences, or alternative perspectives	Occasional asking of clarification and follow-up questions, with limited exploration of causes, consequences, or alternative perspectives	No asking of clarification or follow-up questions
**E. Taking Turns and Responding to Counterarguments**				
E1. Acknowledging opposing views	Clear and respectful recognition and acknowledgment of opposing views before responding	Partial or brief recognition and acknowledgment of opposing views	Mention of opposing views without meaningful engagement	Absence of acknowledgment of opposing views
E2. Defending claims logically	Consistent logical defense of claims using clear reasoning and relevant evidence	Defense mostly logical; minor gaps in reasoning or evidence	Defense weak or partially logical	No defense provided
**F. Making Connections and Drawing Reasoned Conclusions**				

Critical Thinking Skills	Outstanding (4)	Effective (3)	Adequate (2)	Ineffective (1)
F1. Drawing logical conclusions	Conclusions logically follow from discussion and evidence	Conclusions mostly logical; minor gaps	Conclusions weakly connected	Conclusions illogical or absent
F2. Justifying conclusions orally	Clear justification, linking reasons and evidence to the conclusion	Mostly clear justification, with minor gaps in reasoning or evidence	Partial or vague justification, with noticeable gaps	No justification provided

Appendix (C)

Speaking Fluency Skills Scoring Rubric

Speaking Fluency Skills	Outstanding (4)	Effective (3)	Adequate (2)	Ineffective (1)
**A. Speech Flow**				
A1. Initiating continuous speech	Continuous and uninterrupted speech with only brief, natural pauses while ideas are expressed without breakdowns	Speech generally continuous; occasional pauses do not affect message	Frequent stopping; continuity sometimes disrupted	Speech frequently breaks down with long stops
A2. Maintaining appropriate speech rate	Speech rate consistently appropriate; neither rushed nor slow	Speech rate generally appropriate; minor fluctuations	Speech often too slow or too fast	Speech rate consistently inappropriate
**B. Pause Management Fluency**				
B1. Managing silent pauses	Silent pauses are rare and brief (usually **<1 second**) and occur at natural boundaries	Silent pauses occasionally noticeable (**1–2 seconds**) but do not disrupt flow	Silent pauses frequent (**2–4 seconds**) and sometimes interrupt speech	Silent pauses long or frequent (**>4 seconds**), seriously disrupting speech
B2. Managing speech gaps	Purposeful use of filled pauses to maintain the flow of speech	Appropriate but occasionally repetitive use of filled pauses	Frequent use of filled pauses reduces fluency	Excessive reliance on filled pauses
**C. Repair Fluency**				
C1. Self-correction without breakdown	Self-corrections are immediate and smooth without stopping speech	Self-corrections occur with brief hesitation	Self-corrections often interrupt speech	Self-corrections cause breakdowns or stopping

Speaking Fluency Skills	Outstanding (4)	Effective (3)	Adequate (2)	Ineffective (1)
C2. Reformulating ideas smoothly	Clear and smooth reformulation of ideas while maintaining continuous flow	Mostly clear and smooth rephrasing of ideas with minor hesitation	Noticeable disruption in rephrasing of ideas, affecting clarity and flow	Inability to rephrase ideas clearly or maintain smooth flow
C3. Substitution	Consistently substitutes missing words using appropriate synonyms or paraphrases	Frequent substitution of missing words using appropriate synonyms	Limited substitution of missing words using appropriate synonyms	Inability to substitute words, causing speech breakdown
**D. Intonation for Smoothness**				
D1. Using natural intonation patterns	Intonation is natural and consistently supports meaning	Intonation generally appropriate with minor issues	Intonation sometimes unnatural, affecting smoothness	Intonation consistently unnatural or flat
